# Electrographic lead I and V_5_ monitoring could have detected a missed left‐side pneumothorax intraoperatively

**DOI:** 10.1111/anec.13017

**Published:** 2022-11-22

**Authors:** Chihjen Lee, Roya Yumul, Colby Vongchaichinsri, Kevin Tsai, Lena Wang

**Affiliations:** ^1^ Cedars‐Sinai Health System Los Angeles California USA

**Keywords:** electrocardiographic changes, intraoperative monitoring, nephrectomy, pneumothorax

## Abstract

We present an EKG monitoring strategy to detect pneumothorax during high‐risk surgery. In the literature, EKG changes and pneumothorax are well‐described. However, anesthesiologists only monitor lead II on a three‐lead EKG system in the operating room. In our case, there was only a subtle change in lead II for a left‐sided pneumothorax, which could have been easily missed. On the contrary, there was a marked QRS amplitude reduction and T wave flattening/inversion in lead I and V_5_. We recommend lead V_5_ be added to the continuous monitoring and lead I be periodically checked for surgeries known to potentially cause pneumothorax.

## Graphical abstract

Postoperative EKG showed a new right superior axis at 268 degrees and markedly reduced R wave amplitude in lateral leads I and aVL, and precordial leads V _2_ through V _6_. Also noted were T wave flattening/inversion in lead I, lead II and precordial leads Electrical alternans were most visible in V _6_. These EKG findings were consistent with left‐sided pneumothorax. Stat chest X‐ray was ordered, which revealed a large left‐sided pneumothorax and a nearly total collapsed left lung.

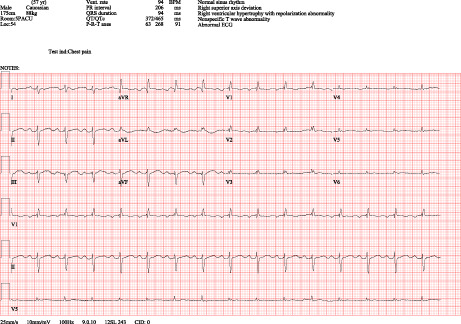

## 1 CASE DESCRIPTION

**FIGURE 1:**
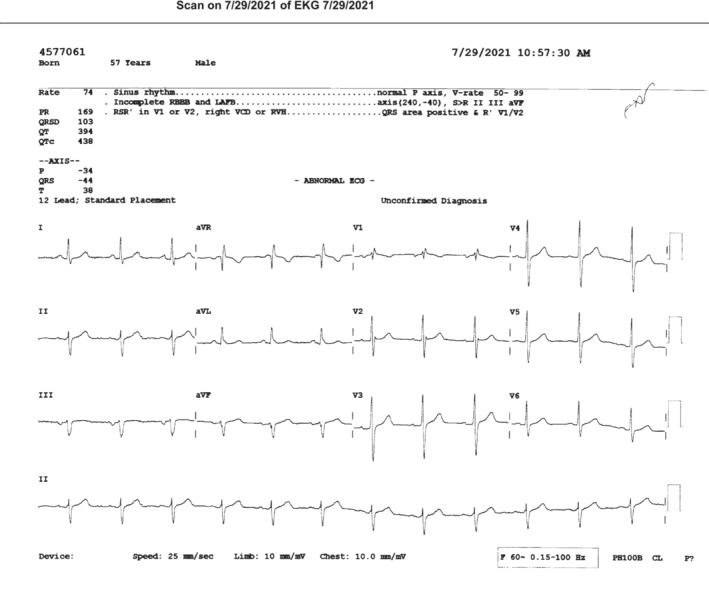
Pre‐op EKG

A 57‐year‐old male patient with a history of thoracic aortic aneurysm and non‐ST elevation myocardial infarction (NSTEMI) 3 months prior, underwent da Vinci robotic partial nephrectomy for a left renal mass. Preoperative laboratories including complete blood count, basic metabolic panel, and coagulation were normal. EKG showed normal sinus rhythm, incomplete right bundle branch block, left anterior fascicular block, and left axis deviation at negative 44 degrees (Figure 1). Angiogram following the last episode of NSTEMI showed normal coronary arteries. Transthoracic echocardiogram showed an ejection fraction of 64%, mild left ventricular hypertrophy, mild‐to‐moderate aortic regurgitation, and normal pulmonary arterial pressure.

Intraoperative course was uneventful. The patient was induced and intubated with fentanyl, Versed, propofol, and rocuronium. Anesthesia was maintained with sevoflurane and propofol infusion. The patient remained paralyzed with rocuronium during surgery. Blood pressure, heart rate, oxygen saturation, end‐tidal CO_2_, and peak airway pressure throughout surgery were all unremarkable. However, upon awakening and extubating the endotracheal tube, the patient became combative and complained of chest pain and shortness of breath. At that time, the patient was breathing 6 L of oxygen via a simple facial mask. Oxygen saturation was 96%. Respiratory rate was 22. Blood pressure was 147/89. Slight tachycardia at 97 was noted. Hydromorphone was initially given for agitation and pain. The patient felt comfortable and fell asleep.

**FIGURE 2:**
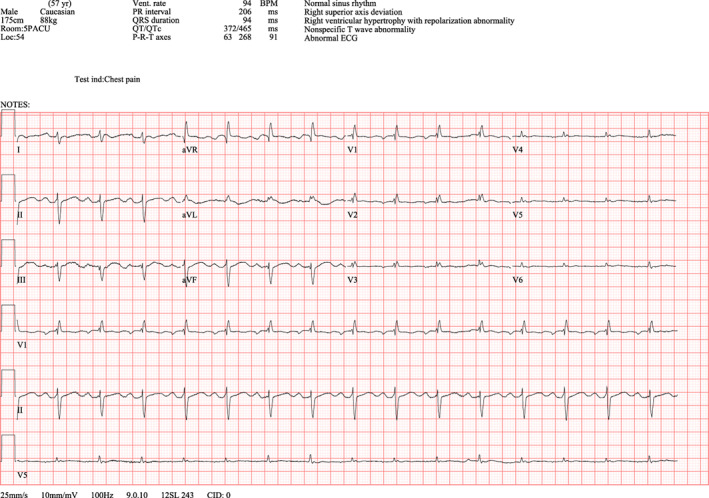
Post‐op EKG

**FIGURE 3:**
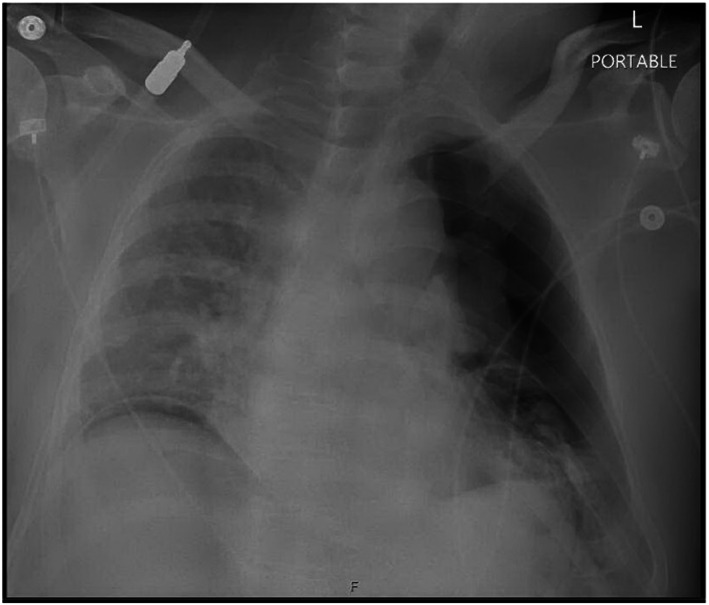
Post‐op CXR

Due to the history of NSTEMI, the acute coronary syndrome was the first to rule out in the recovery room. Postoperative EKG showed a new right superior axis at 268 degrees and markedly reduced R wave amplitude in lateral leads, I and aVL, and precordial leads, V_2_ through V_6_ (Figure 2). Also noted were T wave flattening/inversion in lead I, lead II, and precordial leads. Electrical alternans was most visible in V_6_. These EKG findings were consistent with left‐sided pneumothorax (Kenzaka & Yamazaki, 2016). Stat chest X‐ray was ordered, which revealed a large left‐sided pneumothorax and a nearly total collapsed left lung (Figure 3). Troponin, complete blood count, and basic metabolic panel were normal.

**FIGURE 4:**
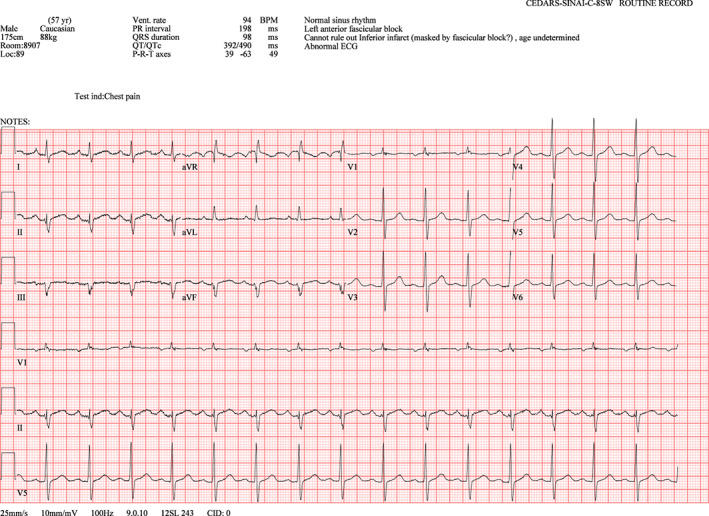
Post chest‐tube EKG

**FIGURE 5:**
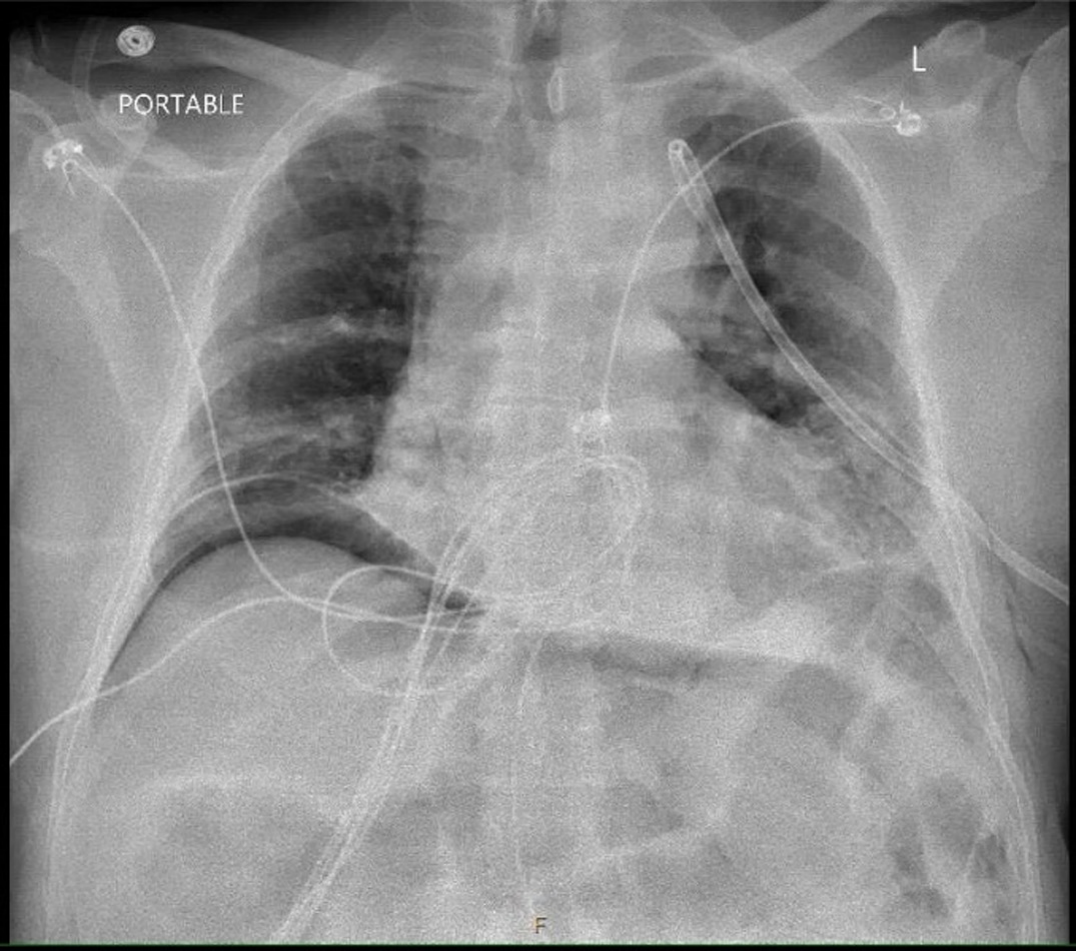
Post chest‐tube CXR

Chest tube was then urgently inserted, and the left lung was re‐expanded. Post chest‐tube EKG (Figure 4) showed restored voltages on lead I, aVL, and V_2_ to V_6_, left anterior fascicular block, and left axis deviation, all of which were similar to the preoperative EKG findings (Figure 1). Repeat chest X‐ray is shown in Figure 5.

## 2 DISCUSSION

Robotic nephrectomy has become a common surgical procedure in recent years. Perioperatively, pneumothorax is a rare but well‐documented complication, which can potentially be life‐threatening if not recognized and treated promptly. However, due to mechanical ventilation and general anesthesia, the patient may not show any signs and symptoms of respiratory distress or hemodynamic changes during surgery. Also, intraoperative changes in blood pressure and heart rate are often attributed to many other factors, such as dehydration, blood loss, surgical stimulation, inadequate anesthesia depth, or side effects of anesthesia medications.

### 2.1 Postoperative chest pain and dyspnea

In our patient, the pneumothorax was not diagnosed until after the surgery when the patient complained of chest pain and dyspnea upon awakening. Given the recent history of NSTEMI, the acute coronary syndrome was the first to be ruled out. Therefore, a 12‐lead EKG was ordered, which did not show ST‐T changes suggestive of cardiac ischemia. Instead, it showed a marked reduction in voltage amplitude on lead I, aVL, and V_2_ through V_6_, suggesting left‐sided pneumothorax (Figure 2). Since only lead II was monitored intraoperatively and it showed only a subtle increase in negative voltage, pneumothorax was never suspected during surgery.

### 2.2 EKG changes and pneumothorax

EKG changes due to pneumothorax have been well‐documented in the literature (Kleine & Roorda, 1976; Klin et al., 2021; Krenke et al., 2008). Depressed amplitudes of QRS complexes and right axis deviation are due to two factors:

1. Increased distance between the heart and the electrodes. The heart is rotated and displaced to the right due to increased intrathoracic pressure.
2. The newly formed air cavity between the heart and the surface electrodes could reduce electrical conductance as air is a poor electrical conductor.

Electrical alternans are QRS amplitude changes associated with respiratory cycles. The heart is displaced more to the left with each inspiration as more air gets into the right lung. V_6_ shows bigger R waves during inspiration and smaller ones during expiration.

In our patient, the voltages on lead I, aVL, and V_2_ through V_6_ were markedly diminished due to the insulating effect of CO_2_ pneumothorax on the left. Of note, the voltage on V_1_ was preserved, since V_1_ lead was placed to the right of the sternum. Our patient also presented with an extreme right superior EKG axis at 268 degrees, which can be explained by the pre‐existing left axis deviation and the newly diminished lateral lead amplitudes. After chest‐tube placement, the amplitudes were dramatically restored, and the EKG returned close to the baseline.

### 2.3 Monitoring other EKG leads

Retrospectively, the EKG changes could have been detected intraoperatively if lead I and lead V_5_ had been monitored. Routinely, anesthesiologists only monitor lead II due to the fact that lead II is the best lead for detecting rhythm abnormalities. Lead V_5_ is sometimes monitored to detect lateral wall ischemia. On the anesthesia monitor, lead I, II, or III can be freely selected on the screen. We suggest that in a patient who is at risk of developing pneumothorax from a particular surgical procedure like robotic nephrectomy, the baseline voltages of lead I, II, and III should be documented and compared periodically during surgery. In addition, lead V_5_ should be monitored continuously.

### 2.4 Right‐sided pneumothorax

In the case of right‐sided surgery, e.g., right nephrectomy, the EKG changes due to right‐sided pneumothorax could be different from the left‐sided pneumothorax. One case report described increased voltages on lead II and flipping of V_5_ polarity from positive to negative (Glenn et al., 1996). A second case reported amplitude diminution in lead I and aVR and flipping of V_3_ polarity from negative to positive (poor progression). Also noted were q waves in lead II, III, and aVF (Tsilakis et al., 2007). Another right pneumothorax also reported amplitude diminution of lead I, and rotation of the heart (namely, upright V_4_ to biphasic V_4_) (Yamamoto et al., 2021). The CO_2_ present in the right chest cavity might displace the heart towards the left chest wall, causing changes in voltage and axis. Rotation of the heart may affect the normal progression of QRS complexes on precordial leads. One common finding in these case reports is diminished voltage in lead I.

### 2.5 Peak airway pressure

Although pneumothorax usually causes an increase in peak airway pressure, change of peak airway pressure can be attributed to many factors: Trendelenburg position, kinking of the anesthesia circuit, kinking of the endotracheal tube, right main stem intubation, and elevation of diaphragms due to CO_2_ insufflation of the abdominal cavity. Therefore, peak airway pressure increase alone is not diagnostic of pneumothorax.

### 2.6 Breath sound

Loss of breath sound on one side of the lung could be a sign of pneumothorax. However, due to the unique operating‐room setting, the lungs could be difficult to auscultate due to the patient positioning, surgical draping, operating‐room music, or noise. In addition, right main stem intubation is a common cause of the loss of the breath sound on the left.

### 2.7 Positioning

Intraoperatively, the EKG monitoring is further complicated by the patient positioning. When the patient is placed in the left lateral decubitus position (left‐side down), the position of the heart could be displaced or rotated. Therefore, the appearance of lead I, II, III, or V_5_ waveforms could differ from that of the preoperative 12 lead EKG (Adams & Drew, 1997). It is important to document the new baseline EKG appearance after the position change. Our patient was placed in the right lateral decubitus position (right‐side down). Because of the support of the mediastinum, the position of the heart and the appearance of EKG waveforms did not change much. Any deviation from the new baseline after the positioning, either right or left lateral decubitus, should trigger an investigation accordingly.

### 2.8 Point of care ultrasound

Lastly, Point of Care Ultrasound (POCUS) has become an invaluable tool for anesthesiologists. It is portable, noninvasive, and readily available in the operating‐room setting. Loss of lung sliding and pulsing is a sensitive indicator for pneumothorax; the presence of lung point confirms the diagnosis.

In conclusion, for surgeries with a high risk of developing pneumothorax, we suggest anesthesiologists monitor lead V_5_ continuously and/or check lead I and lead III periodically. Voltage diminution in Lead I, and polarity flipping in precordial leads could be the first sign of pneumothorax.

## AUTHOR CONTRIBUTIONS

Chihjen Lee contributed to conceptualization, data acquisition and analysis, literature search, manuscript preparation, editing, revision and proofreading. Roya Yumul contributed to conceptualization and manuscript preparation. Colby Vongchaichinsri contributed to manuscript preparation, editing and revision. Kevin Tsai contributed to data acquisition and analysis. Lena Wang contributed to conceptualization, data acquisition and analysis, literature search, manuscript preparation, editing, revision and proofreading.

## CONFLICT OF INTEREST

No conflict of interest.

## ETHICAL APPROVAL

Not applicable.

## PATIENT CONSENT STATEMENT

A written consent for publication was obtained from the patient.

## Data Availability

No data available.
